# CCAAT/enhancer binding protein beta protects muscle satellite cells from apoptosis after injury and in cancer cachexia

**DOI:** 10.1038/cddis.2016.4

**Published:** 2016-02-25

**Authors:** F Marchildon, D Fu, N Lala-Tabbert, N Wiper-Bergeron

**Affiliations:** 1 Graduate Program in Cellular and Molecular Medicine, Faculty of Medicine, University of Ottawa, Ottawa, Ontario, Canada; 2 Department of Cellular and Molecular Medicine, Faculty of Medicine, University of Ottawa, Ottawa, Ontario, Canada

## Abstract

CCAAT/enhancer binding protein beta (C/EBP*β*), a transcription factor expressed in muscle satellite cells (SCs), inhibits the myogenic program and is downregulated early in differentiation. In a conditional null model in which C/EBP*β* expression is knocked down in paired box protein 7+ (Pax7+) SCs, cardiotoxin (CTX) injury is poorly repaired, although muscle regeneration is efficient in control littermates. While myoblasts lacking C/EBP*β* can differentiate efficiently in culture, after CTX injury poor regeneration was attributed to a smaller than normal Pax7+ population, which was not due to a failure of SCs to proliferate. Rather, the percentage of apoptotic SCs was increased in muscle lacking C/EBP*β*. Given that an injury induced by BaCl_2_ is repaired with greater efficiency than controls in the absence of C/EBP*β*, we investigated the inflammatory response following BaCl_2_ and CTX injury and found that the levels of interleukin-1*β* (IL-1*β*), a proinflammatory cytokine, were robustly elevated following CTX injury and could induce C/EBP*β* expression in myoblasts. High levels of C/EBP*β* expression in myoblasts correlated with resistance to apoptotic stimuli, while its loss increased sensitivity to thapsigargin-induced cell death. Using cancer cachexia as a model for chronic inflammation, we found that C/EBP*β* expression was increased in SCs and myoblasts of tumor-bearing cachectic animals. Further, in cachectic conditional knockout animals lacking C/EBP*β* in Pax7+ cells, the SC compartment was reduced because of increased apoptosis, and regeneration was impaired. Our findings indicate that the stimulation of C/EBP*β* expression by IL-1*β* following muscle injury and in cancer cachexia acts to promote SC survival, and is therefore a protective mechanism for SCs and myoblasts in the face of inflammation.

Efficient regeneration of skeletal muscle is dependent on tissue-resident stem cells termed satellite cells (SCs).^1^ Quiescent and heterogeneous by nature, SCs are characterized by their histological localization between the muscle fiber sarcolemma and the basal lamina, as well as by expression of the paired box protein 7 (Pax7).^2, 3^ Upon muscle injury, Pax7 expression declines and activated SCs progressively stimulate the expression of the myogenic basic helix-loop-helix regulatory factors that are required for the induction of myocyte-specific genes.^4^

CCAAT/enhancer binding proteins (C/EBPs) are a family of bZIP transcription factors involved in numerous biological processes.^5, 6, 7, 8, 9^ *Cebpb* null mice have defective liver regeneration,^10^ skin abnormalities,^11^ impaired development of the mammary glands,^12^ reduced adipose tissue,^13^ female sterility^14^ and are immunodeficient.^15, 16, 17^ In healthy skeletal muscle, C/EBP*β* expression is restricted to Pax7^+^ SCs. Highest in SCs, C/EBP*β* levels decline early in differentiation and this downregulation is required for myogenesis to occur.^18, 19^ Indeed, forcing C/EBP*β* expression in myoblasts blocks myogenesis, and is accompanied by increased Pax7 and decreased myogenic differentiation factor 1 (MyoD), myogenin and myosin heavy chain expression. In addition, loss of C/EBP*β* in SCs results in larger myotubes in culture, muscle hypertrophy *in vivo* and enhanced muscle regeneration following a single BaCl_2_-induced injury.^19^

Normal skeletal muscle repair involves local inflammation that is required for efficient regeneration. M1-type macrophages are recruited early to the site of injury, produce interleukin-1 (IL-1), IL-6 and tumor necrosis factor *α* (TNF*α*), and promote SC proliferation while inhibiting their differentiation.^20, 21^ Four days after injury, M2-type macrophages become the dominant subtype in the muscle and act to decrease local inflammation by deactivating M1 macrophages.^22, 23^ The transient inflammatory response following acute muscle injury is accompanied by an increase in Pax7+ cells in the injured muscle that do not immediately contribute to repair.^24, 25, 26^ Resolution of inflammation, rather, promotes myogenesis.^27, 28, 29, 30^ Although acute muscle injury is accompanied by transient inflammation, chronic inflammation and dysregulated cytokine production is a feature of cachexia, characterized by both adipose tissue and skeletal muscle atrophy, and occurs in many ailments including chronic obstructive pulmonary disease, AIDS, chronic kidney failure and sepsis.^31, 32, 33, 34^

Relatively little is known about the effect of inflammation on muscle stem cell populations after acute injury, and even less under chronic conditions such as cachexia. Herein, we describe a protective mechanism where IL-1*β* production after acute muscle injury or in cachexia drives increased C/EBP*β* expression in muscle SCs, rendering SCs more resistant to apoptosis. Loss of C/EBP*β* expression triggers the loss of Pax7^+^ cells by apoptosis and impairs muscle regeneration.

## Results

### CTX injury increases apoptosis of Cebpb null SCs

**Figure 1:**
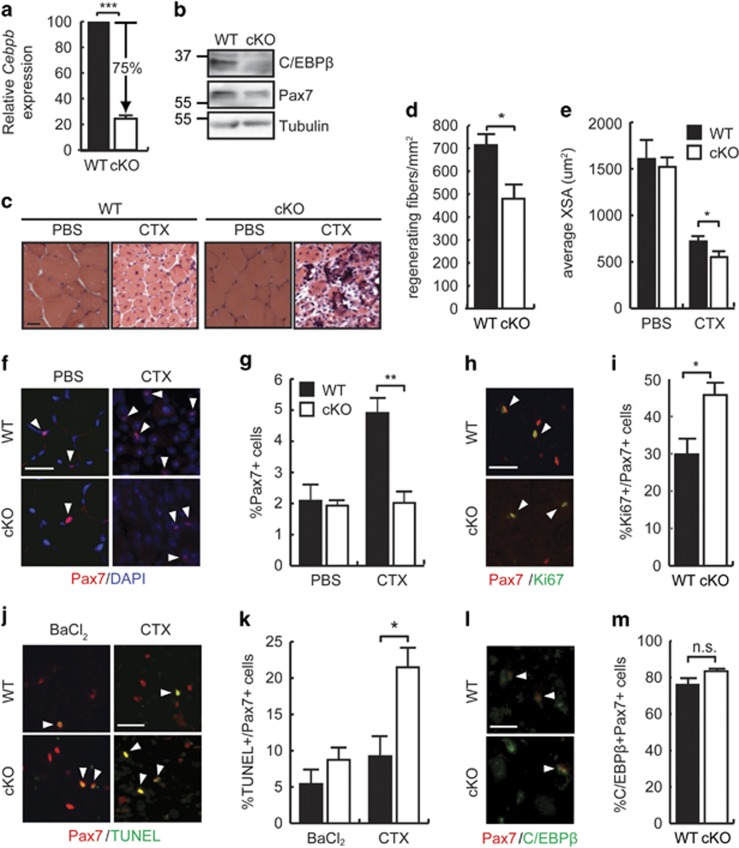
C/EBP*β* protects muscle SCs from apoptosis following CTX injury. (**a**) Relative *Cebpb* mRNA expression in myoblasts isolated from WT (*Cebpb*^*fl/fl*^*Pax7*^*+/+*^) and cKO (*Cebpb*^*fl/fl*^*Pax7*^*CreERT/+*^) uninjured hind limb muscles, normalized to 18 S. Both groups received IP tamoxifen treatment to induce *Cebpb* excision in Cre-expressing animals. ***P=1 × 10^−8^, *n*=4. (**b**) C/EBP*β* and Pax7 protein expression in primary myoblasts isolated from mice treated as in (**a**). Cyclophilin B (CyPB) is a loading control. (**c**) H&E-stained TA muscle cross-sections from female WT and cKO mice 7 days after injury with CTX or sham injured with PBS. Scale bar=20 *μ*m. (**d**) Number of regenerating muscle fibers per mm^2^ in TA muscle from WT or cKO mice 7 days post-injury with CTX. *P<0.05, *n*=3. (**e**) Average fiber cross-sectional area (XSA) 7 days after injury with CTX or sham injury (PBS) in WT and cKO TA muscle. *P<0.05, *n*=4. (**f**) Pax7 immunostaining of WT and cKO TA muscle 7 days post sham (PBS) or CTX injury. Scale bar=20 *μ*m. Arrowheads indicate positively stained cells. (**g**) Percentage of Pax7+ cells (relative to total DAPI+ nuclei) found in TA muscle from (**f**). **P=0.010, *n*=3. (**h**) Representative images of TUNEL and Pax7 immunostaining of TA muscle 4 days after injury with BaCl_2_. Scale bar=20 *μ*m. (**i**) Percentage of Ki67/Pax7+ cells relative to total Pax7+ cells in the TA of WT and cKO mice from (**h**). *P<0.05, *n*=3 (**j**) Representative images of immunostaining for Pax7 and TUNEL staining on WT and cKO TA muscle 4 days after injury with BaCl_2_ or CTX. Scale bar=20 *μ*m. (**k**) Percentage of TUNEL+/Pax7+ cells (relative to total Pax7+ cells) in TA of WT and cKO as in (**j**). *P<0.05, *n*=3. (**l**) Representative images of immunostaining for Pax7 and C/EBP*β* on TA muscle from WT and cKO 7 days after injury with CTX. Scale bar=20 *μ*m. (**m**) Percentage of C/EBP*β*+/Pax7+ cells (relative to total Pax7+ cells) in TA muscle as in (**l**). NS, not significant

C/EBP*β* conditional knockout mice (cKO, *Cebpb*^*fl/fl*^*Pax7*^*+/CreER*^), where *Cebpb* is excised in Pax7^+^ cells, and non-Cre-expressing littermate controls (WT, *Cebpb*^*fl/fl*^*Pax7*^*+/+*^) were injured with cardiotoxin (CTX) and repair was assessed 7 days post-injury. An excision efficiency of approximately 75% was achieved in primary myoblasts isolated from cKO mice as compared with WT, and this correlated with a decrease in C/EBP*β* protein expression in these same cells (Figures 1a and b). In sharp contrast to BaCl_2_-induced injury, where cKOs repaired the damage with greater efficiency than WT controls,^19^ after CTX injury, cKOs failed to appreciably repair muscle (Figure 1c). The number of fibers with centrally located nuclei, indicating regeneration, was decreased by approximately 33% in cKOs after CTX injury as compared with controls with an average cross-sectional area ~24% smaller than WT (Figures 1d and e). Given that SCs lacking C/EBP*β* can differentiate efficiently *in vitro,*^19^ we reasoned that the CTX injury may be affecting the size of the SC population in cKO mice. Although uninjured WT and cKO muscle had equivalent percentages of Pax7^+^ cells (~2% of total nuclei), injury increased this value approximately twofold in WT mice (Figures 1f and g). However, the increase in the Pax7^+^ population was not observed in the cKOs after injury, but rather remained at uninjured levels. The smaller Pax7^+^ population in the cKOs was not because of a failure of these cells to proliferate, as the percentage of Pax7+ cells that were also Ki67+ was greater in cKO cultures 7 days after injury with BaCl_2_ as compared with WT muscle (Figures 1h and i). Rather, CTX injury provoked a ~2.5-fold increase in the number of terminal deoxynucleotidyl transferase dUTP nick end labeling (TUNEL)^+^/Pax7^+^ cells in cKOs after CTX injury when compared with WT mice, but not after BaCl_2_-induced injury, indicating that CTX injury promotes the apoptosis of SCs lacking C/EBP*β* (Figures 1j and k). Interestingly, the remaining SCs in cKO muscle after injury with CTX were mostly C/EBP*β* positive, suggesting that they are likely 'recombination escapers' (Figures 1l and m).

### C/EBP*β* is upregulated by IL-1*β*

**Figure 2:**
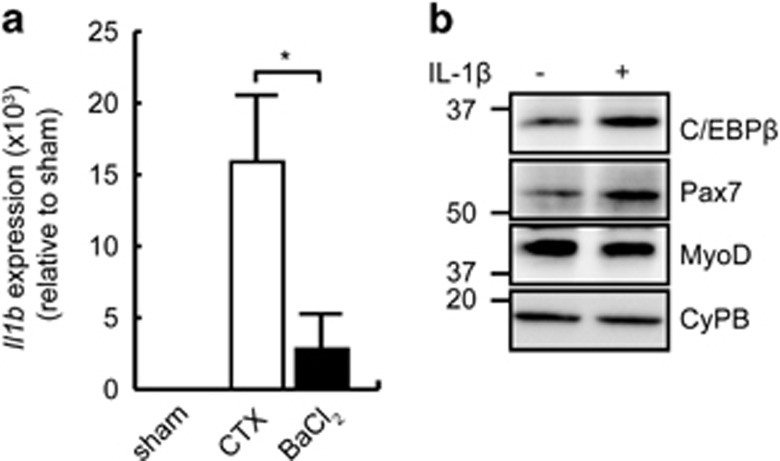
Il-1*β* upregulates C/EBP*β* expression. (**a**) *Il1b* expression in whole TA muscle 24 h after injury with CTX or BaCl_2_. Sham injury was performed with PBS. *n*=5, *P<0.05. (**b**) Western analysis of C/EBP*β*, Pax7 and MyoD protein expression in primary myoblasts incubated with IL-1*β* for 24 h in growth medium. The migration of molecular weight markers, in kDa, is shown. Cyclophilin B (CyPB) is a loading control

Following injury to the skeletal muscle, recruited leukocytes express and secrete a constellation of cytokines that act in a paracrine manner in the injured micro-environment.^21, 35^ Given the contrast in the regenerative response to CTX and BaCl_2_ in cKO animals, we hypothesized that the two injuries generate a different immune response. Among cytokines measured, IL-1*β* expression, which was not detected in uninjured muscle, was ~6-fold higher in CTX-injured muscles than after BaCl_2_ injury (Figure 2a). C/EBP*β* levels after treatment with IL-1*β* were next assessed in primary myoblasts (Figure 2b). C/EBP*β* expression was upregulated following exposure to IL-1*β*, and concomitant with the higher C/EBP*β*, we observed an increase in Pax7 expression and reduced MyoD protein levels as previously reported.^19^

### C/EBP*β* protects myoblasts from apoptosis

**Figure 3:**
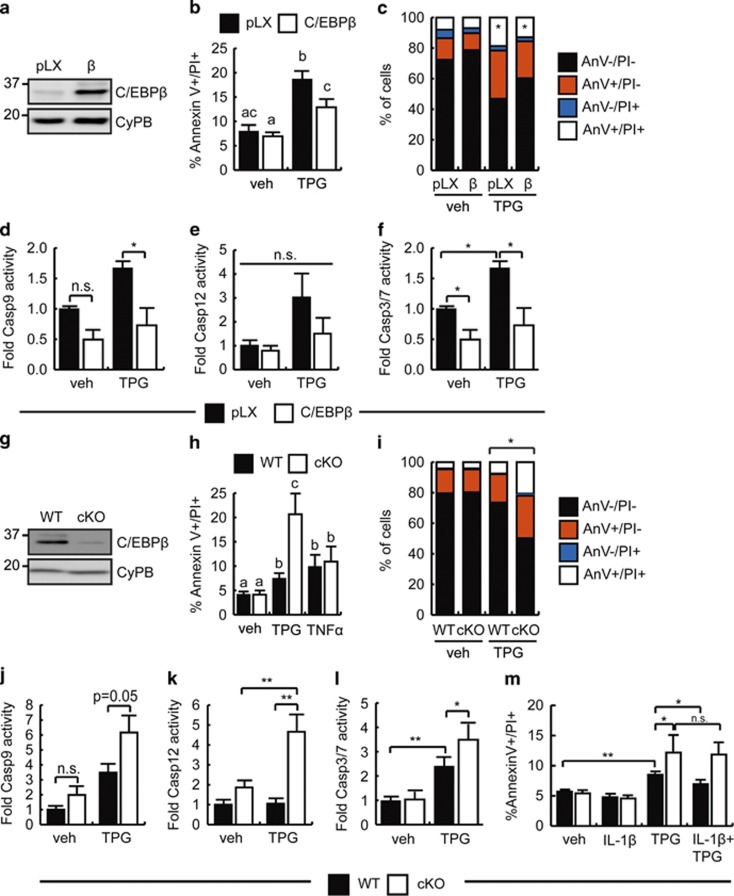
C/EBP*β* promotes the survival of myoblasts. (**a**) C/EBP*β* protein expression in proliferating C2C12 cells retrovirally transduced to express C/EBP*β* or with empty virus (pLXSN). The migration of molecular weight markers, in kDa, is shown. Cyclophilin B (CyPB) is a loading control. (**b**) Percentage of dead cells determined by flow cytometry analysis of Annexin V and PI staining in vehicle and TPG-treated C2C12 stable cells. Means indicated with different letters are significantly different from one another, *n*=4. Gating strategy and sample plots are provided in Supplementary Figure S2. (**c**) Percentage of cells found in the Annexin V+/PI+, Annexin V+/PI–, Annexin V–/PI– and Annexin V+/PI+ populations from cells treated as in (**b**). Two populations indicated with an asterisk are significantly different from one another. Gating strategy and sample plots are provided in Supplementary Figure S2. (**d**) Caspase-9 activation in vehicle and TPG-treated C2C12 stable cell lines treated as in (**b**), shown relative to vehicle-treated empty virus control cells. NS, nonsignificant, *P<0.05, *n*=3. (**e**) Caspase-12 activation in vehicle and TPG-treated C2C12 stable cell lines treated as in (**b**), shown relative to vehicle-treated empty virus control cells. *n*=6. (**f**) Caspase-3/7 activity in TPG-treated C2C12-overexpressing C/EBP*β* shown relative to C2C12 empty vector controls. *P<0.05, *n*=4. (**g**) Western analysis of C/EBP*β* protein expression in primary myoblasts from WT and cKO mice treated with 4-OH tamoxifen for 48 h to induce excision. (**h**) Percentage of dead cells determined by flow cytometry in vehicle and TPG-treated WT and cKO myoblasts. Means indicated with different letters are significantly different from one another, *n*=6. Gating strategy and sample plots are provided in Supplementary Figure S3. (**i**) Percentage of cells found in the Annexin V+/PI+, Annexin V+/PI–, Annexin V–/PI– and Annexin V+/PI+ populations from cells treated as in (**h**). Matching populations in the cell lines indicated with an asterisk are significantly different from one another. Gating strategy and sample plots are provided in Supplementary Figure S3. (**j**) Caspase-9 activation in vehicle and TPG-treated primary myoblasts cell lines treated as in (**h**), shown relative to vehicle-treated WT cells. *n*=3. (**k**) Caspase-12 activation in vehicle and TPG-treated primary myoblasts treated as in (**h**), shown relative to vehicle-treated WT control cells. **P<0.01, *n*=3. (**l**) Caspase-3/7 activity in TPG-treated cKO myoblasts relative to WT. *P<0.05, **P<0.01, *n*=4. (**m**) Percentage of dead cells determined by flow cytometry in WT and cKO myoblasts pretreated with IL-1*β* and TPG for 24 hrs, as indicated. *P<0.05, **P<0.01, *n*=3. Gating strategy and sample plots are provided in Supplementary Figure S4

Quiescent SCs are known to be resistant to apoptosis,^36, 37, 38^ although following activation, myoblasts become increasingly sensitive to apoptotic signals in a time frame concomitant with decreasing C/EBP*β* levels.^19, 39^ Indeed, C/EBP*β* has previously been implicated in cell survival.^40, 41, 42^ Given the increase in apoptotic SCs lacking C/EBP*β* after CTX injury, we tested if C/EBP*β* could promote myoblast survival. C2C12 myoblasts were retrovirally transduced to express C/EBP*β* or with empty virus (pLXSN) and pooled stable cell lines were treated with thapsigargin (TPG) to trigger apoptosis (Figures 3a and b). TPG promotes ER stress and results in activation of caspase-9 and/or caspase-12 by inhibiting the sarcoplasmic reticulum calcium ATPase SERCA.^43, 44, 45^ In the absence of treatment, approximately 7% of empty virus control and C/EBP*β*-overexpressing myoblasts cells were dead by apoptosis, defined as being both propidium iodide-positive (PI+) and Annexin V-positive (Figure 3b). TPG treatment stimulated a twofold increase in dead cells in empty virus controls to approximately 20%, whereas ectopic expression of C/EBP*β* protected against TPG-induced apoptosis, with an PI+/Annexin V+ population of ~12% that was not statistically different from controls. Analysis of the PI+, Annexin V+ and double-negative populations revealed that while TPG increased the Annexin V–/PI+ and Annexin V+/PI+ populations in both empty virus controls and C/EBP*β* overexpressors, the only significant difference found between cell types was the percentage of dead cells (indicated by the asterisk) (Figure 3c).

Activity of the initiator caspase-9, which has been implicated in the apoptotic pathway downstream of TPG, was significantly reduced in C/EBP*β*-overexpressing cells after TPG treatment, and was also reduced in vehicle-treated cells although not meeting statistical significance (Figure 3d). Caspase-12 activity, although trending toward an increase following TPG treatment in empty virus control cells, was highly variable and failed to change significantly in any of the conditions tested (Figure 3e). Consistent with the reduction in dead cells observed, caspase 3/7 activity was decreased 66% in TPG-treated C/EBP*β*-overexpressing myoblasts as compared with empty virus controls (Figure 3f).

In primary myoblasts, treatment with TPG resulted in an almost twofold increase in dead cells in WT cultures to approximately 7.5% (Figure 3h). In primary myoblasts lacking C/EBP*β*, the PI+/Annexin V+ population increased to ~20% following TPG treatment, 2.7-fold more dead cells than TPG-treated WT cultures (Figure 3h). Interestingly, the effect was not generalizable to other apoptotic stimuli. Although TNF*α* treatment could significantly increase the percentage of dead WT cells, this effect was not magnified with loss of C/EBP*β*, suggesting that C/EBP*β*'s anti-apoptotic effects may be limited to intrinsic ER stress-mediated pathways (Figure 3h). Following TPG treatment, cKO cultures had significantly more dead (Annexin V+/PI+), Annexin V+/PI– and Annexin V–/PI+ cells than WT controls, and significantly fewer Annexin V–/PI– cells (Figure 3i). In agreement with our gain-of-function data, loss of C/EBP*β* expression resulted in increased activation of caspase-9 following TPG treatment, as compared with WT controls (Figure 3j). Further, although TPG treatment did not stimulate caspase-12 activation in WT cultures, caspase-12 activity was significantly increased in cKO cultures following TPG treatment (Figure 3k), suggesting that C/EBP*β* may act to negatively regulate caspase-12 activity. In accordance with these results, caspase 3/7 activity was significantly increased by 50% in TPG-treated cKO cells as compared with WT (Figure 3l). Taken together, these data indicate that C/EBP*β* regulates myoblast sensitivity to apoptosis.

Given that IL-1*β* can stimulate C/EBP*β* expression, we asked whether treatment with IL-1*β* could protect myoblasts from TPG-induced apoptosis in a C/EBP*β*-dependent manner. In WT primary myoblasts, treatment with IL-1*β* alone did not increase the percentage of PI+/Annexin V+ cells as compared with vehicle-treated cultures (Figure 3m). Although treatment with TPG increased the percentage of WT cells dying, when IL-1*β* was added before TPG treatment to upregulate C/EBP*β*, the percentage of PI+/Annexin V+ cells decreased significantly (Figure 3m). This protective effect was lost in cKO myoblasts, in which IL-1*β* failed to significantly reduce the population of dead cells following TPG treatment. Analysis of the Annexin V+, PI+ and double-negative populations revealed that the Annexin V–/PI– population was significantly larger in WT myoblasts treated with IL-1*β* and TPG as compared with cKO cells while the double-positive population was significantly smaller, consistent with higher levels of apoptosis in the cKO cultures that was not rescued by IL-1*β* treatment (Supplementary Figure S1).

### Cancer cachexia increases C/EBP*β* expression in SCs

**Figure 4:**
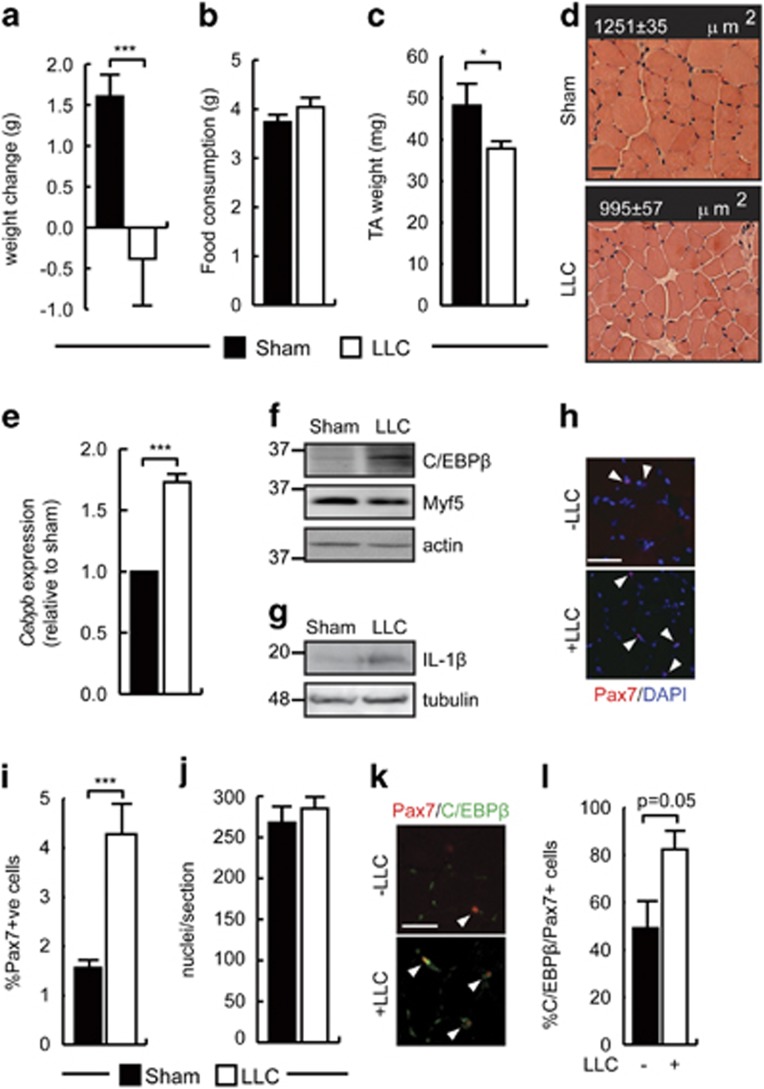
Cancer cachexia increases C/EBP*β* expression in SCs and inhibits myogenesis. (**a**) Average weight gain of female C57BL/6 sham and LLC-injected mice (8 weeks old) 4 weeks after grafting. ***P=0.001, *n*=15. (**b**) Average daily food consumption of sham and LLC tumor grafted animals. (**c**) Average TA mass in healthy and cachectic mice as in (**a**). *P<0.05, *n*=5. (**d**) H&E-stained TA cross-sections from sham and LLC-bearing mice 4 weeks after grafting. Mean fiber cross-sectional areas (XSA) are indicated±S.E.M.. *P*=0.006, *n*=5. Scale bar=20 *μ*m. (**e)** *Cebpb* expression in primary myoblasts isolated from healthy and cachectic mice. Freshly isolated cells were plated at equal densities and expanded in culture for 3 days without passaging before analysis. ***P=0.001, *n*=6. (**f**) C/EBP*β* expression in primary myoblasts isolated from healthy and LLC-bearing mice. Freshly isolated cells were plated at equal densities and expanded in culture for 3 days without passaging before analysis. Actin is a loading control. (**g**) IL-1*β* expression in TA muscle of sham and LLC-bearing mice. The migration of molecular weight markers, in kDa, is shown. Tubulin is a loading control. (**h**) Representative images of immunohistochemistry for Pax7 expression in TA muscle isolated from mice as in (**a**). Scale bar=50 *μ*m. Arrowheads indicate positively stained cells. (**i**) Percentage of Pax7+ cells (relative to total nuclei) in TA muscle isolated from mice as in (**a**). ***P<0.001, *n*=6. (**j**) Quantification of nuclei per section of TA muscle as in (**i**). (**k**) Representative images of Pax7 and C/EBP*β* immunostaining on TA muscle sections from animals treated as in (**a**). Scale bar=20 *μ*m. Arrowheads indicate double-positive stained cells. (**l**) Percentage of C/EBP*β*/Pax7+ cells (relative to total Pax7+ cells) in TA muscle from healthy and cachectic mice from (**k**). *n*>3

High levels of proinflammatory cytokines including IL-1*β* are associated with muscle wasting and cachexia. To assess whether chronic inflammation could regulate C/EBP*β* expression in myoblasts *in vivo*, cancer cachexia was induced in mice using the Lewis lung carcinoma (LLC) syngenic tumor graft model.^46, 47^ LLC cells were transplanted into mice, which developed tumors (with a mean mass of 2.43±0.31 g) and cachexia within 4 weeks of grafting. At necropsy, sham-injected animals had gained on average 1.6 g, whereas LLC-injected animals lost 0.4 g while maintaining normal appetites (Figures 4a and b). At necropsy, tibialis anterior (TA) mass was reduced approximately 20% in the LLC-bearing animals (Figure 4c) and histological analysis of muscle cross-sections revealed a 20% decrease in the average fiber area in LLC-injected animals as compared with sham controls, supporting the development of cachexia (Figure 4d).

Isolation of primary myoblasts from healthy and tumor-bearing animals revealed a ~1.5-fold increase in *Cebpb* expression in cachectic animals and higher levels of C/EBP*β* protein (Figures 4e and f). These cells were not passaged following removal from the muscle, but were cultured in normal growth medium for 3 days before analysis, suggesting that the induction of C/EBP*β* by the cachectic environment could persist *ex vivo*. The expression of IL-1*β* was also increased in the whole muscle from cachectic animals as compared with sham controls (Figure 4g). Further, immunohistochemical analysis of the SC population in cachectic mice revealed that the Pax7+ population increased more than twofold in the TA muscles of LLC-bearing animals as compared with sham controls, without differences in the total number of nuclei, indicating that SCs are present in greater numbers in cachectic mice (Figures 4I and j) consistent with previous observations.^48^ Further, the proportion of C/EBP*β*^+^/Pax7^+^ cells was increased in the cachectic animals as compared with sham controls, suggesting that larger Pax7+ population observed in cachectic animals was largely also C/EBP*β*-expressing. (Figures 4k and l).

### C/EBP*β* is required for SCs expansion in cancer cachexia

**Figure 5:**
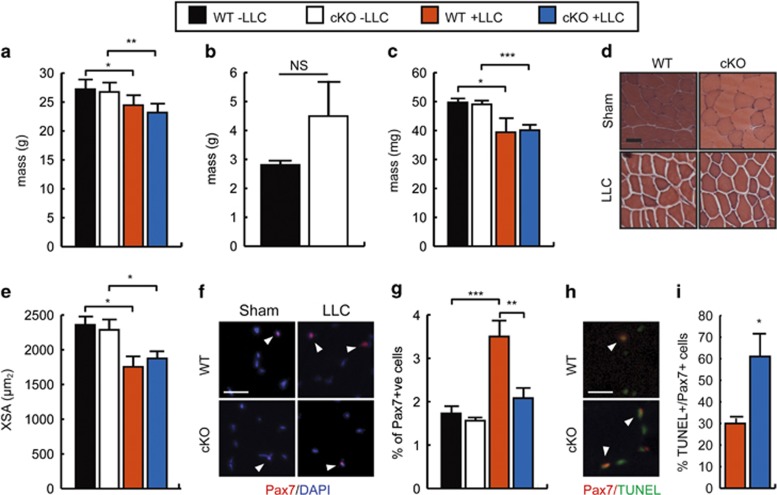
Smaller SC population in cancer cachexia with loss of C/EBP*β*. (**a**) Average body weight of WT and cKO mice 4 weeks after sham or LLC tumor graft. Eight-week-old WT and cKO male mice in the C57BL/6 genetic background received IP tamoxifen injections 5 days before tumor graft. *P<0.05, **P<0.01, *n*=5. (**b**) Tumor mass at necropsy in WT and cKO animals as in (**a**). (**c**) TA mass in WT and cKO sham or LLC-bearing mice. *P<0.05 and ***P<0.001, *n*=5. (**d**) Representative images of TA cross-sections from WT and cKO sham and LLC-bearing mice. Scale bar=50 *μ*m. (**e**) Average TA fiber cross-sectional area (XSA) in WT and cKO sham or LLC-bearing mice. *P<0.05, *n*=4. (**f**) Representative images of immunostaining for Pax7 expression in WT and cKO sham and LLC-bearing mice. Scale bar=20 *μ*m. Arrowheads indicate positively stained cells. (**g**) Percentage of Pax7+ cells (relative to total DAPI+ nuclei) in WT and cKO sham and LLC-bearing mice. **P<0.01, ***P<0.001, *n*=10. (**h**) Representative images of immunostaining for Pax7+ and TUNEL+ cells in TA muscle of WT and cKO sham and LLC-bearing mice. Scale bar=20 *μ*m. Arrowheads indicate double-positive cells. (**i**) Percentage of apoptotic Pax7+ cells (TUNEL+/Pax7+ cells relative to total Pax7+ cells) found in TA muscle of WT and cKO sham and LLC-bearing mice. *P<0.05, *n*=3

To determine whether loss of C/EBP*β* could sensitize SCs to apoptosis in the context of cancer cachexia, we grafted the LLC tumor into cKO animals (Figure 5). Although there was no difference in body weight between sham-injected WT and cKO animals in the absence of tumor, both WT and cKO animals lost approximately 10% of their body weight following tumor graft, indicative of cachexia (Figure 5a). Tumor mass was equivalent in both genotypes, although more variable in the cKOs (Figure 5b). Cachexia was accompanied by a decrease in TA weight in both WT and cKOs, although no significant differences were noted between WT and cKO tumor-bearing animals (Figure 5c). Similarly, although the average fiber cross-sectional area was decreased significantly in both genotypes with cachexia, the cKO animals were comparable to WT cachectic animals, suggesting that loss of C/EBP*β* expression in SCs did not affect muscle wasting in cancer cachexia (Figures 5d and e).

Examination of the number of Pax7^+^ cells in uninjured WT and cKO muscle from healthy and LLC-grafted mice revealed that while the population of Pax7^+^ SCs in both healthy WT and cKO TA muscles were comparable, cachexia expanded the Pax7^+^ population from 1.7 to 3.5% in WT animals, but not in cKO animals bearing the LLC tumor (Figures 5f and g). Further, cKO animals with cachexia had an ~2-fold increase in the percentage of TUNEL+/Pax7+ cells compared with WT tumor-bearing animals, indicating that the smaller Pax7^+^ population found in cKO animals is also mostly apoptotic and suggesting that C/EBP*β* may acts to protect SCs from apoptosis in the context of chronic inflammation typical of cachexia (Figures 5h and i). TUNEL staining was undetectable in healthy controls of both genotypes.

**Figure 6:**
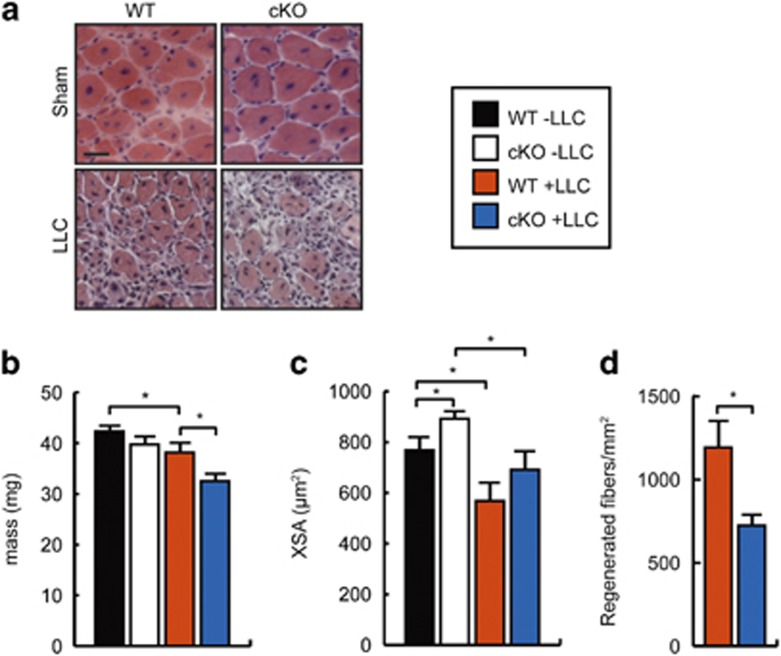
Inhibition of regeneration in cachectic mice lacking C/EBP*β* in Pax7+ cells. (**a**) H&E-stained TA cross-sections from sham and LLC-grafted WT or cKO mice 7 days after BaCl_2_ injury. Scale bar=20 *μ*m. (**b**) Average TA mass in sham or LLC-grafted WT or cKO animals 7 days after BaCl_2_ injury. *P<0.05, *n*=5. (**c**) Average TA fiber cross-sectional area (XSA) in sham or LLC-grafted BaCl_2_-injured WT or cKO mice. *P<0.05, *n*=5. (**d**) Number of regenerating muscle fibers per mm^2^ 7 days after BaCl_2_ injury to the TA in LLC-grafted WT and cKO mice. *P<0.05, *n*=5

To determine if the smaller Pax7^+^ population in cachectic cKO animals was able to sustain muscle regeneration, we injured both healthy and cachectic WT and cKO mice with BaCl_2_, as this myotoxin does not on its own trigger apoptosis in the absence of cachexia. After injury, cKO tumor-bearing animals failed to repair as efficiently as WT cachectic controls (Figure 6a). Indeed, although both healthy WT and cKO animals restored TA weight similarly after injury, the TA mass of WT LLC-bearing animals was reduced by ~10% of controls, whereas in cachectic cKO animals it was decreased ~18% from healthy controls and significantly less that the tumor-bearing WT cohort, suggesting that loss of C/EBP*β* further impairs regeneration in cachectic animals (Figure 6b). Although injury with BaCl_2_ resulted in larger myofibers after injury as previously reported, the average regenerating fiber cross-sectional area after injury in tumor-bearing WT and cKO mice was reduced ~25% as compared with healthy controls, with no significant differences between genotypes (Figure 6c). However, the number of regenerating fibers was reduced by ~37% in cKO animals as compared with WT controls indicating that the repair defect is limited to the number of regenerating fibers and not their size (Figure 6d). Taken together, these results suggest that loss of C/EBP*β* can exacerbate the regeneration defect in cachectic mice.

## Discussion

SCs and myotubes are known to be relatively resistant to apoptotic stimuli.^36, 49^ Injury and subsequent activation of SCs increase their vulnerability to apoptosis. However, the mechanism by which SCs withstand apoptosis is poorly defined. Pax7 expression is considered protective from apoptosis, as deletion of Pax7 triggers cell cycle abnormalities characterized by an extended G2/M phase, and a progressive loss of muscle precursors to cell death.^50, 51^ Recently, Brg1, a component of the Swi/Snf chromatin remodeling complex, was shown to be required for maintaining viability in myoblasts, and this through regulation of Pax7 expression.^52^ Interestingly, Pax7 is also a target of C/EBP*β* in proliferating myoblasts and in differentiating cultures.^19^ Induction of C/EBP*β* by IL-1*β* stimulates Pax7 expression and thus, C/EBP*β* may act through Pax7 to protect muscle SCs from apoptosis. In addition, Myod1^−/−^ myoblasts are resistant to apoptosis both *in vitro* and *in vivo* and C/EBP*β* is a potent inhibitor of MyoD protein expression, making it straightforward to speculate that C/EBP*β* could also modulate sensitivity to apoptosis through the control of MyoD expression.^53^ C/EBP*β* can also directly inhibit caspase activity, and regulate p53 activity and expression, both of which could also regulate sensitivity to apoptotic signals.^41, 42^ Our results suggest that C/EBP*β* may also negatively regulate caspase-12.

In acute injury, when the inflammation is short-lived, regeneration is restrained and resolution of the inflammation would be expected to reduce C/EBP*β* expression allowing for the initiation of myogenesis.^27, 28, 29, 30^ The decrease in inflammation concomitant with a decrease C/EBP*β* expression is indeed consistent with the known time frame of myoblast differentiation after injury.^29^ In chronic inflammation, the levels of proinflammatory cytokines remain high, leading to an expansion of the Pax7^+^ population, but a defect in muscle repair. Loss of C/EBP*β* expression in the context of cancer cachexia does not exacerbate muscle wasting, but does decrease the SC compartment through apoptosis, and impairs muscle regeneration. It remains unknown whether the SC numbers and activity recover when the inflammatory milieu resolves, but given that tumor resection can improve cachexia, it is likely that the persistence of C/EBP*β* in muscle SCs in the cachectic animal is transient.^48^ In our experiments, we can, however, detect an increase in C/EBP*β* expression up to a week after removal from the cachectic milieu. As such, the induction of C/EBP*β* in SCs may act as a sensor for inflammation providing both survival signals and concomitantly a blockade of regeneration.

Although a pro-survival role for C/EBP*β* has been described in the development of cancer (hepatocellular carcinoma and melanomas), our results identify a function for C/EBP*β* in an adult stem cell population. In *gallus gallus*, C/EBP*β* (NF-M) promotes survival in hematopoietic progenitor cells, suggesting that our findings could extend to a broad range of stem cell populations as well as a diverse range of organisms.^54^

One of the limitations of murine cachexia models is the relatively short term in which muscle wasting is studied. Unlike human cachexia, the wasting in mice persists for only a few weeks before the cachexia is too profound for humane treatment. As such, it is impossible to know from our experiments, in which the animals were killed at approximately 10% weight loss with relatively mild cachexia, whether the induction of C/EBP*β* expression by the cachectic environment persists as cachexia worsens or whether the cells eventually become desensitized to the effects of proinflammatory cytokines. In the case of cytokine resistance, we would expect loss of C/EBP*β* expression in cachexia to trigger apoptosis of activated muscle precursors resulting in loss of the regenerative response and a more rapid loss of muscle mass. Indeed, an increase in SC apoptosis in a cachexia model with more severe weight loss has been observed.^55^ Moreover, muscle biopsies from patients suffering of gastrointestinal cancers had increased DNA fragmentation, typical of apoptosis, suggesting that loss of muscle cells contributes to wasting in humans.^56^ These findings would suggest that anti-inflammatory therapy could release the C/EBP*β*-imposed blockade of muscle repair and therefore counteract the loss of muscle protein observed in cachexia.^57^

## Materials and Methods

### Animal models

All animal handling procedures conformed to the guidelines established by the University of Ottawa Animal Care Service and the Canadian Council on Animal Care. Mice carrying a C/EBP*β*-floxed allele^58^ and the mouse carrying the Pax7-CreER^tm^ allele^59^ were maintained in a mixed genetic background, to generate control (WT, *Cebpb*^*fl/fl*^*Pax7*^*+/+*^) and conditional null (cKO, *Cebpb*^*fl/fl*^*Pax*^*CreER/+*^) mice. Young WT and cKO littermates received daily intraperitoneal (IP) injections of tamoxifen (2 mg/20 g) for 5 days to excise *Cebpb*. To allow growth of LLC cells in transgenic animals, C/EBP*β*^fl/fl^ and Pax7^CreER/+^ mice were backcrossed to C57BL/6 (Jackson, Bar Harbor, ME, USA) mice for nine generations. For all LLC experiment, 6- to 8-week-old mice were used. For the induction of cancer cachexia, 5x10^5^ sub-confluent LLC cells washed in PBS were injected subcutaneously while PBS alone was used for sham animals.

### Cell culture and differentiation

C2C12 cells (ATCC, Manassas, VA, USA) were maintained in DMEM supplemented with 10% fetal bovine serum (FBS). Primary WT and cKO myoblasts cultures, obtained by enzymatic digestion and pre-plating as previously described,^19^ were maintained in DMEM supplemented with 20% FBS, 10% horse serum (HS), 10 ng/ml basic fibroblast growth factor and 2ng/ml hepatocyte growth factor (Peprotech, Rocky Hill, NJ, USA). For isolation of primary myoblasts from cachectic animals, cells were seeded onto plates after purification and were allowed to expand in growth medium without passaging. *In vitro* activation of the Cre DNA recombinase was done by addition of 2 *μ*M 4-OH tamoxifen (Sigma, Oakville, ON, Canada). Primary myoblasts were stimulated to differentiate in DMEM supplemented with 10% HS and 2% FBS. Replication-incompetent retroviruses were produced by calcium phosphate transfection of the Phoenix packaging cell line. Infection of C2C12 myoblasts was perform as described.^60^ The pLXSN-C/EBP*β* plasmid has been described previously.^61^ LLC cells were maintained in DMEM supplemented with 10% FBS. All cell lines were kept in a humidified atmosphere at 37 °C with 5% CO_2_.

### TA muscle injury

For injuries, 30 *μ*l of CTX (Latoxan, Valence, France) at 10^−5^ M or 50 *μ*l of 1.2% BaCl_2_ (Sigma) both dissolved in PBS were injected into the left TA muscle using a 28 1/2 gauge syringe. PBS was injected in the right TA muscle for control. At necropsy, TA muscle was dissected and fixed in 10% formalin and paraffin embedded or flash frozen in melting isopentane.

### Immunocytochemistry, immunohistochemistry and TUNEL

*In situ* TUNEL assays were performed according to the manufacturer's instructions (Roche, Laval, QC, Canada) and counter-stained with DAPI (0.5 *μ*g/ml) for 5 min. For immunohistochemistry, muscle sections were air-dried 30 min at 65 °C and fixed 10 min in 4% paraformaldehyde. After washes, antigen retrieval was done for 20 min at 92 °C with citrate buffer (10 mM citric acid, 0.05% Tween-20, pH 6.0) and sections were cooled to 20 °C. Sections were permeabilized 10 min in 0.5% Triton X-100, washed and blocked 1 h in 5% normal donkey serum (Jackson Immunoresearch, West Grove, PA, USA). Antibodies used for detection were: mouse anti-Pax7 (DSHB, 1/100), and rabbit anti-C/EBP*β* (Santa Cruz Biotechnology, Dallas, TX, USA, SC-150, 1/100) biotin-conjugated donkey anti-mouse IgG (Jackson Immunoresearch) with Cy3-strepavidin (Jackson Immunoresearch), and Alexa488-conjugated donkey anti-rabbit IgG (Jackson Immunoresearch). All primary antibodies were added on sections and incubated at 4 °C overnight. Secondary antibody labeling was done for 1 h.

### Reverse-transcriptase quantitative PCR

Total RNA was purified at indicated times with the RNAeasy kit (Qiagen, Germantown, MD, USA). In all, 1 *μ*g of purified RNA was DNase digested for 1 h at 37 °C (Ambion, Burlington, ON, Canada) and cDNA was synthesized using the iScript kit (Bio-Rad, Mississauga, ON, Canada). cDNA was PCR amplified on a Stratagene MX3005p real-time thermocycler (Agilent Technologies, Mississauga, Ontario, Canada) using a iTaq universal SYBR Green kit (Bio-Rad). Relative transcript expression was computed using the ΔΔCt method.

### Caspase assays, Annexin V and PI staining

Primary myoblasts were incubated with IL-1*β* (Sigma) at 20 ng/ml for 24 h starting 6 h before the addition of 100 nM TPG (Sigma) to trigger apoptosis. Caspase activity was assessed following the manufacturer's instructions (caspase-3/7 (Promega, Madison, WI, USA), caspase-9, caspase-12 (Abcam, Toronto, ON, Canada). For assessment of apoptosis by flow cytometry, myoblasts were collected by trypsin, washed in ice-cold PBS and resuspended in Annexin V buffer (10 mM HEPES, 140 mM NaCl, 2.5 mM CaCl_2_, pH 7.4) and labeled with Annexin V and PI according to the manufacturer's instructions (Life Technologies, Burlington, ON, Canada). Cells were analyzed by flow cytometry on a Beckman CyAn ADP instrument (Beckman Coulter Canada, Mississauga, Ontario, Canada) and dot plots were made and analyzed with Kaluza software (Mississauga, ON, Canada).

### Microscope acquisition and imaging

For histological images, muscle sections were pictured with brightfield light microscope CX42 (Olympus, Richmond Hill, ON, Canada) using a Qcapture 3 camera (Surrey, BC, Canada). Representative images are presented and were equally processed by Photoshop (Adobe) by adjusting levels uniformly. For fluorescent images, pictures were taken on a DMI3000B (Leica, Concord, ON, Canada) epifluorescence microscope using infinity 3 (Lumenera, Ottawa, ON, Canada) camera. For processing, individual pictures were level adjusted uniformly and pasted on a single color channel in Photoshop.

### Antibodies and western blot

Whole-cell lysate were prepared in IPH buffer (50 mM Tris pH 7.5, 150 mM NaCl, 0.5% NP-40, 5 mM EDTA, 1 mM DTT and 1X protease inhibitor cocktail) and briefly sonicated on ice. For detection, the following antibodies were used: mouse anti-Myod1 (SC-32758), rabbit anti-C/EBP*β* (SC-150) rabbit anti-Myf5 (SC-302), rabbit anti-tubulin (SC-5286) from Santa Cruz Biotechnology, mouse anti-Pax7 (DSHB), rabbit anti-IL-1*β* and rabbit anti-cyclophilin B from Abcam and mouse anti-*β*-actin (Sigma).

### Statistical analysis, sample size, randomization and blinding

For bar graphs, data are represented as the mean±S.E.M. For statistical analysis, two samples were subjected to a two-tailed Student's *t*-test assuming equal variance and normal distribution. Different populations were identified as having a *P*-value <0.05. When analyzing data with more than two groups, an ANOVA with *post-hoc* test was used. All *in vitro* experiments were performed on a minimum of three independent trials. All *in vivo* experiments were performed on a minimum of five animals per group.

## Abbreviations

C/EBP*β*: CCAAT/enhancer binding protein beta
Pax7: paired box protein 7
IL-1*β*: interleukin-1 beta
SC: satellite cell
MyoD: myogenic differentiation factor 1
cKO: conditional knockout
CTX: cardiotoxin
TUNEL: terminal deoxynucleotidyl transferase dUTP nick end labeling
TPG: thapsigargin
PI: propidium iodine
LLC: Lewis lung carcinoma
