# Supplementary material for: CCAAT/enhancer binding protein beta protects muscle satellite cells from apoptosis after injury and in cancer cachexia
Source: Cell Death Dis. 2016 Feb 25;7(2):e2109–. doi: 10.1038/cddis.2016.4 (PMC4849162; doi:10.1038/cddis.2016.4)
Supplement: Supplementary Figure Legends [file cddis20164x1.docx]

**Supplementary Figure Legends**

**Figure S1. C/EBPβ is required for IL-1β-mediated protection from apoptosis.** Primary myoblasts from WT and cKO muscle were treated with IL-1β for 6 hours after which TPG was added for a total of 24 hours. Percentage of cells found in the Annexin V+/PI+, Annexin V+/PI-, AnnexinV-/PI- and AnnexinV+/PI+ populations from cells treated was determined by flow cytometry. Populations indicated by the asterisk are significantly different from one another. Populations designated with the # are also significantly different from one another (p<0.05).

**Figure S2. Gating strategy and representative data for flow cytometric analysis of C2C12 cells overexpressing C/EBPβ.** **(A)** C2C12 cells retrovirally transduced to express C/EBPβ or with empty virus (pLXSN) were treated with thapsigargin (TPG) or vehicle for 24 hours in growth medium. Forward scatter (FS) and side scatter (SS) dot blot of empty virus control cells (pLXSN) treated with vehicle is shown. **(B)** Linear SS versus SS area plot was used to exclude doublets from analysis of cells as in (A). **(C)** Histogram of Alexa488 fluorescence in pLXSN control cells treated with vehicle, with gate “D” representing cells considered Annexin V positive. **(D)** Histogram of PI fluorescence in pLXSN control cells treated with vehicle where Gate E defines PI-positive cells. **(E)** Representative dot plots for indicated treatment conditions. C- = cells considered negative for both Annexin V and PI fluorescence, C+- = cells positive for PI fluorescence, C-+=cells positive for Annexin V fluorescence and C++ =cells positive for both PI and Annexin V fluorescence.

**Figure S3. Gating strategy and representative data for flow cytometric analysis of primary myoblasts.** **(A)** Primary myoblasts isolated from conditional null mice in which C/EBPβ was deleted in Pax7+ cells (cKO) and littermate controls (WT) were treated with thapsigargin (TPG), Tumor Necrosis Factor α (TNFα) or vehicle for 24 hours in growth medium. Forward scatter (FS) and side scatter (SS) dot blot of a sample run of WT cells treated with vehicle is shown. **(B)** Linear SS versus SS area plot was used to gate to exclude doublets from cells as in (A). **(C)** Histogram of Alexa488 fluorescence in WT cells treated with vehicle, with gate “B” representing cells considered Annexin V positive. **(D)** Histogram of PI fluorescence in WT cells treated with vehicle where Gate D defines PI-positive cells. **(E)** Representative dot plots for indicated treatment conditions. C- = cells considered negative for both Annexin V and PI fluorescence, C+- = cells positive for PI fluorescence, C-+=cells positive for Annexin V fluorescence and C++ = cells positive for both PI and Annexin V fluorescence.

**Figure S4. Gating strategy and representative data for flow cytometric analysis of primary myoblast pretreated with IL-1β.** **(A)** Primary myoblasts from WT and cKO mice were treated with IL-1β or vehicle for 6 hours and then treated with thapsigargin (TPG) or vehicle for a total of 24 hours in growth medium. Forward scatter (FS) and side scatter (SS) dot blot of a sample run of WT treated with vehicle alone is shown. **(B)** Linear SS versus SS area plot was used to gate to exclude doublets from cells as in (A). **(C)** Histogram of Alexa488 fluorescence in WT cells treated with vehicle, with gate “D” representing cells considered Annexin V positive. **(D)** Histogram of PI fluorescence in WT cells treated with vehicle where Gate B defines PI-positive cells. **(E)** Representative dot plots for indicated treatment conditions. C- = cells considered negative for both Annexin V and PI fluorescence, C+- = Cells positive for PI fluorescence, C-+=cells positive for Annexin V fluorescence and C++ = cells positive for both PI and Annexin V fluorescence.
