# Supplementary figures and images for: CCAAT/enhancer binding protein beta protects muscle satellite cells from apoptosis after injury and in cancer cachexia
Source: Cell Death Dis. 2016 Feb 25;7(2):e2109–. doi: 10.1038/cddis.2016.4 (PMC4849162; doi:10.1038/cddis.2016.4)

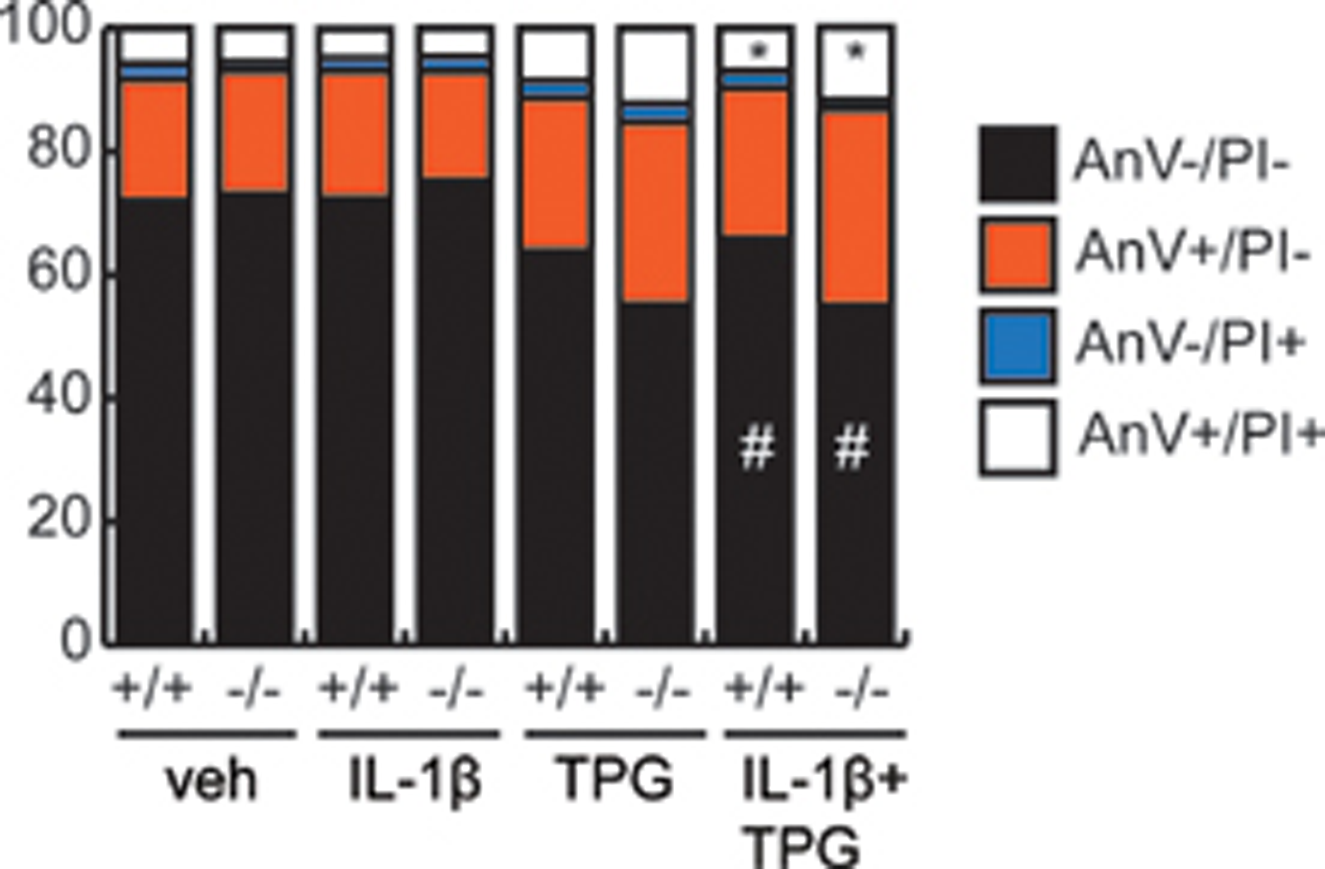

Supplement: Supplementary Figure 1 [file cddis20164x2.tif]

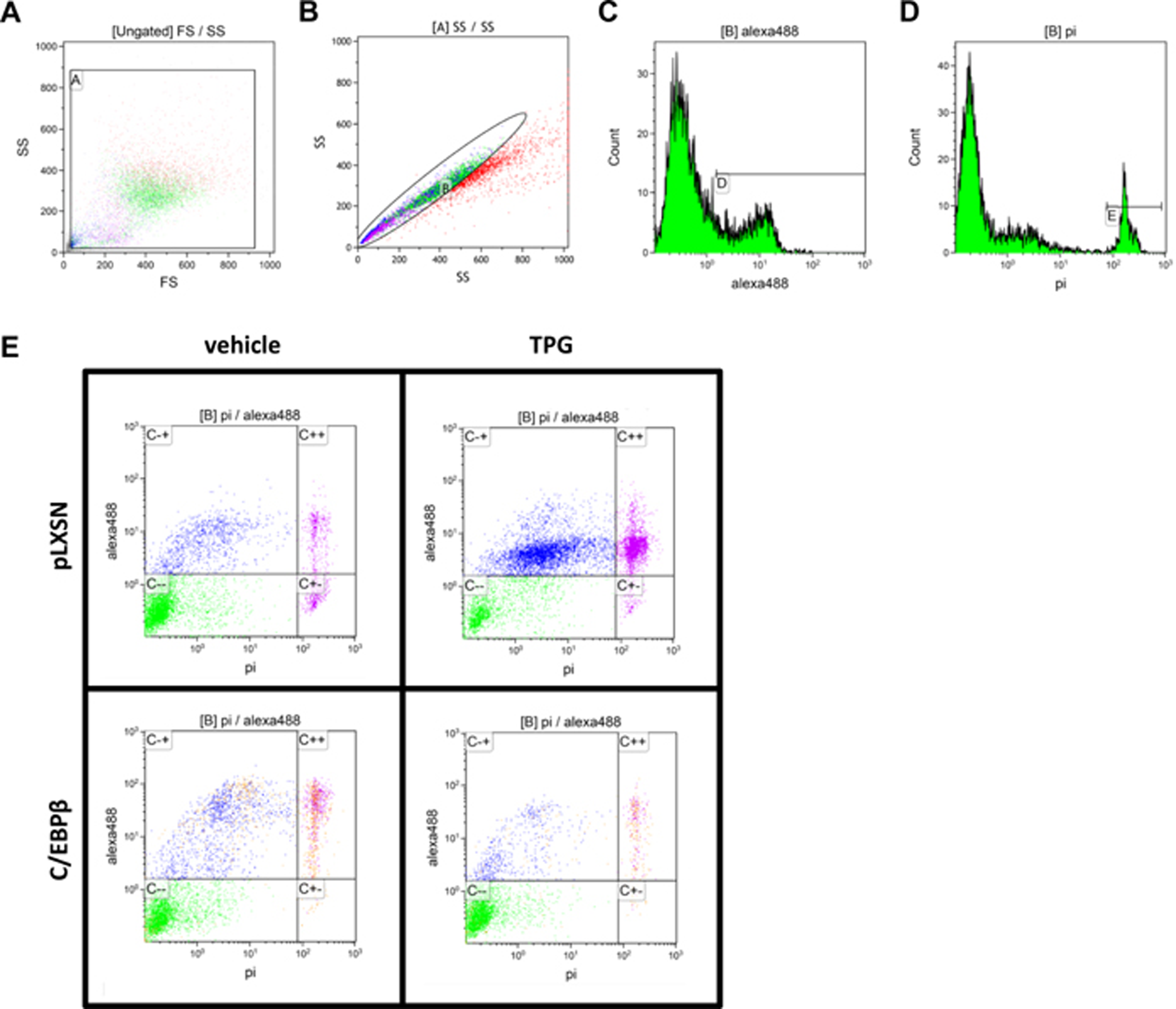

Supplement: Supplementary Figure 2 [file cddis20164x3.tif]

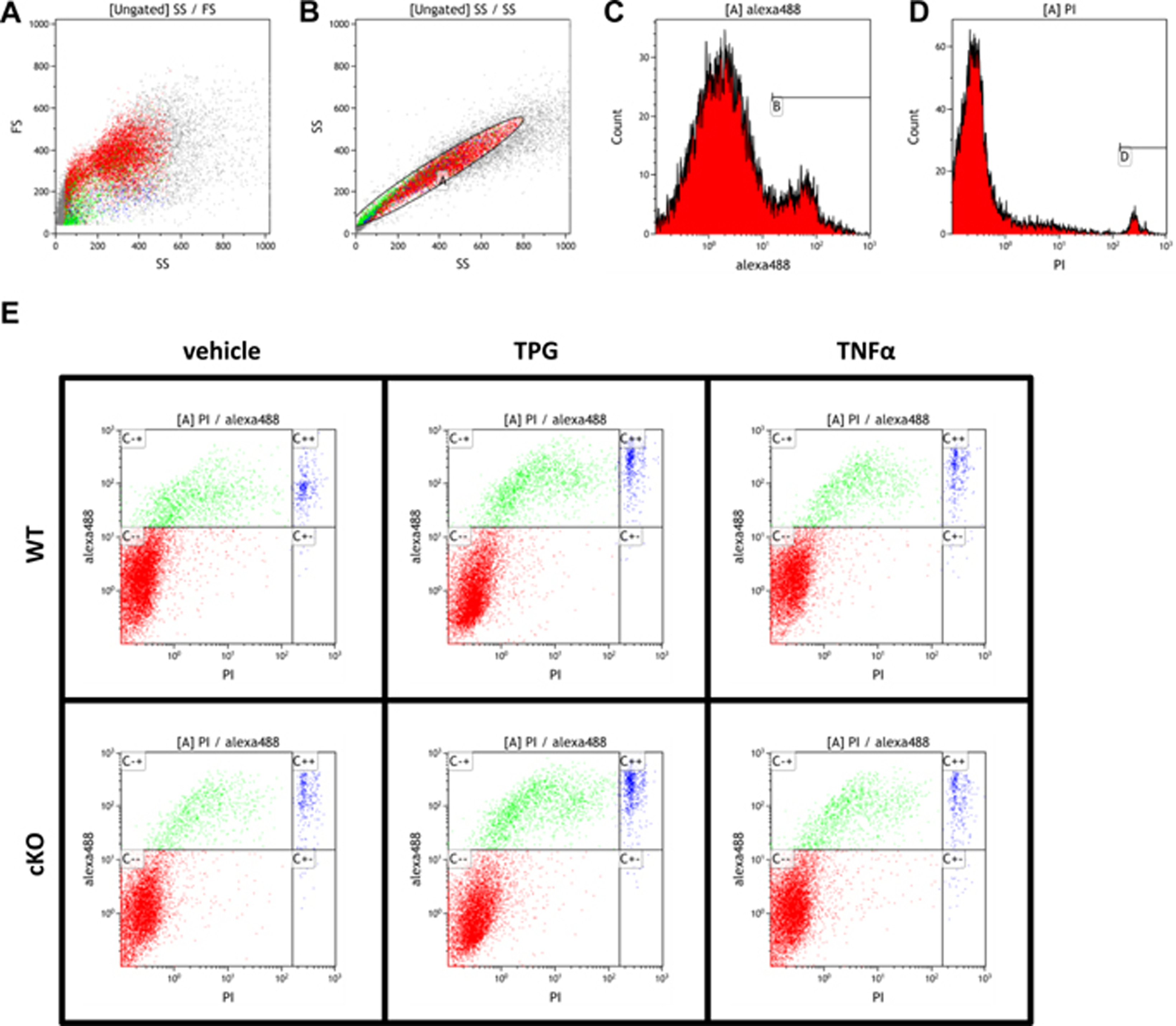

Supplement: Supplementary Figure 3 [file cddis20164x4.tif]

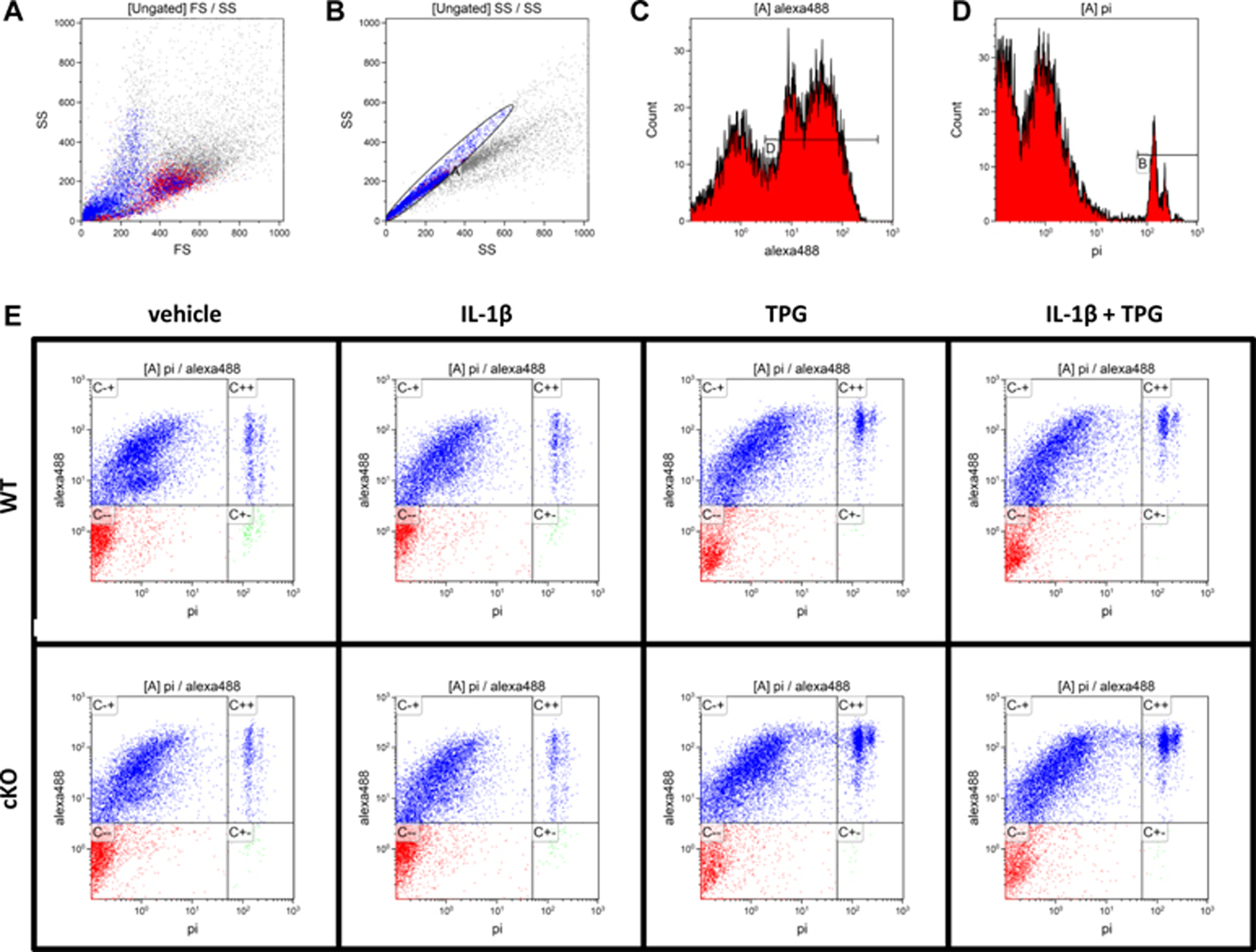

Supplement: Supplementary Figure 4 [file cddis20164x5.tif]
